# Iodothyronine Deiodinase 3 Gene Expression in Gastrointestinal Stromal Tumors: A Pilot Study to Contribute to Risk Assessment

**DOI:** 10.7759/cureus.67426

**Published:** 2024-08-21

**Authors:** Melinda Kolcsár, Ivett-Adrienn Zeces, Attila Kövecsi, Zsolt Kovács, Zsolt Gáll

**Affiliations:** 1 Department of Pharmacology and Clinical Pharmacy, George Emil Palade University of Medicine, Pharmacy, Science, and Technology of Târgu Mureș, Târgu Mureș, ROU; 2 Department of Endocrinology, Târgu Mureş County Emergency Clinical Hospital, Târgu Mureș, ROU; 3 Faculty of Medicine, George Emil Palade University of Medicine, Pharmacy, Science, and Technology of Târgu Mureș, Târgu Mureș, ROU; 4 Department of Pathology, George Emil Palade University of Medicine, Pharmacy, Science, and Technology of Târgu Mureș, Targu Mures, ROU; 5 Department of Pathology, Târgu Mureş County Emergency Clinical Hospital, Târgu Mureș, ROU; 6 Department of Biochemistry and Environmental Chemistry, George Emil Palade University of Medicine, Pharmacy, Science, and Technology of Târgu Mureș, Târgu Mureș, ROU; 7 Department of Pathology, Research Center of Oncopathology and Translational Research, Târgu Mureș, ROU

**Keywords:** precision therapy, consumptive hypothyroidism, tumor prognostic, gastrointestinal stromal tumor, deiodinase type 3

## Abstract

Background: In the realm of gastrointestinal stromal tumors (GIST), understanding the molecular landscape and prognostic factors is crucial for effective management. The deiodinase 3 gene (DIO3), known for its role in thyroid hormone regulation and cell proliferation, has emerged as a potential player in GIST pathogenesis. Our study investigated DIO3 expression in GIST samples and its correlation with tumor characteristics, aiming to enhance prognostic stratification and personalized treatment strategies.

Materials and methods: Using a retrospective design, we analyzed data and formalin-fixed paraffin-embedded (FFPE) samples of patients diagnosed with GIST. The study cohort comprised 33 patients, predominantly female, with a median age of 66 years. The tumor characteristics were meticulously documented, including location, size, mitotic count, risk classification, and immunohistochemical markers. Gene expression analysis of DIO3 was conducted using FFPE samples, with a focus on relative quantification and association with immunohistochemical markers and prognostic risk.

Results: DIO3 overexpression was observed in 69.70% of tumors, while underexpression was noted in 30.30% of cases. Association analyses revealed intriguing insights. A notable association was identified between DIO3 expression and the frequency of DOG1, suggesting a potential interplay between these markers in GIST pathobiology. Furthermore, increased DIO3 expression was significantly higher in very low/low-risk prognostic patients, hinting at a possible link between DIO3 levels and tumor progression prognosis.

Conclusions: The intricate interplay between DIO3 expression and GIST characteristics uncovered in this study underscores the potential of molecular markers in refining prognostic assessments and therapeutic strategies for GIST patients.

## Introduction

Iodothyronine deiodinase 3 (DIO3), the gene encoding the thyroid hormone-inactivating enzyme type 3 deiodinase, is expressed in various physiological and pathological conditions. Studies have highlighted that DIO3 is highly expressed in fetal tissues, such as the placenta, neonatal skin, skeletal muscle, and the central nervous system, where it plays a crucial role in inactivating intracellular T3 [1]. DIO3 has an important role in facilitating cell proliferation and physiological growth [2]. Additionally, DIO3 expression has been associated with seasonal adaptations, with its expression levels changing in response to photoperiod variations, suggesting a role in physiological adaptations triggered by changes in day length [3,4]. The increased expression of DIO3 has been observed in pathological hyperproliferative conditions, where it has been implicated in cell proliferation and differentiation [5,6].

Furthermore, DIO3 expression has been linked to skeletal muscle regeneration, where it is detected in myogenic stem cells and macrophages post-injury [7]. In the context of thyroid carcinoma, increased expression of DIO3 has been reported, suggesting a potential role in this thyroid-stimulating hormone (TSH)-dependent pathological condition [8]. In various other cancers, DIO3 has been implicated through its association with the imprinted delta-like non-canonical Notch ligand 1 (DLK1)-DIO3 locus. Aberrant expression of microRNAs located within this region has been linked to the pathogenesis of several tumors, including esophageal squamous cell carcinoma, gastric cancer, gastrointestinal stromal tumor (GIST), and colorectal cancer [9]. Moreover, the DLK1-DIO3 locus, containing maternally expressed non-coding RNA (ncRNA) genes (MEG) like the lncRNA MEG3 and a cluster of over 50 miRNAs, has been implicated in modulating stemness in embryonic stem cells and cancer progression [10]. Additionally, miRNAs from the DLK1-DIO3 cluster have been described as tumor suppressors in different cancer types, suggesting their potential role in cancer progression [11]. In contrast, low DIO3 expression, possibly caused by gene hypermethylation, was associated with reduced overall survival in breast cancer patients [12]. The dysregulation of DIO3 and its associated ncRNAs within this locus highlights their potential as diagnostic markers and therapeutic targets in cancer management [13].

Gastrointestinal stromal tumors, representing 0.2% of gastrointestinal cancers, start in very early forms of specialized cells in the walls of the gastrointestinal tract called the interstitial cells of Cajal. They are primarily caused by activating mutations in the KIT (CD117, a class III receptor tyrosine kinase) gene, which encodes the platelet-derived growth factor receptor-alpha (PDGFRA), a tyrosine kinase protein [14]. GISTs present a complex scenario in terms of prognostic factors. Several studies have highlighted key factors that play a crucial role in determining the prognosis of patients with GISTs, the tumor size, mitotic index, epithelioid or mixed cellularity, intestinal location, tumor rupture, intraperitoneal hemorrhage, multifocality, and incomplete resection being recognized as significant prognostic factors [15]. Additionally, the expression of oncoprotein c-myc, proliferative index, KIT and PDGFRA mutational status, Ki-67, and circulating tumor DNA (ctDNA) mutation type have been identified as important prognostic indicators in GISTs [16-19].

In very low and low-risk GISTs, adjuvant treatment with tyrosine kinase inhibitors (TKIs) may not be necessary, as these tumors have a lower risk of recurrence and may not benefit from the potential side effects of such therapy. On the other hand, intermediate and high-risk GISTs, especially those with mutations sensitive to imatinib, often require adjuvant treatment with TKIs to improve outcomes and reduce the risk of recurrence. The decision to use TKIs in GIST patients is typically based on the risk profile of the tumor and the potential benefits of adjuvant therapy in preventing disease progression and improving survival rates [17].

Considering the various factors that influence the prognosis of GISTs, our pilot, small sample size study was designed to examine the expression of the DIO3 gene in GIST and its potential correlations with tumor characteristics, aiming to contribute valuable insights to the field and potentially improve the management of GISTs.

## Materials and methods

Study population

In this retrospective study, we used data and formalin-fixed paraffin-embedded (FFPE) samples from patients diagnosed with GIST at the Department of Pathology of Târgu Mureș County Emergency Clinical Hospital between 2015 and 2020. The study received approval from the Ethics Committee at George Emil Palade University of Medicine, Pharmacy, Science, and Technology of Târgu Mureș, with approval number 33468/29.12.2022. The inclusion criteria were the post-surgery histopathological diagnosis of primary, unifocal, non-treated GISTs including CD117, CD34, and DOG1 immunohistochemistry analysis. The exclusion criteria were previously treated GISTs, use of TKIs for other reasons, multifocal or syndromic GISTs, and extra-gastrointestinal location or metastatic GIST at the origin of the tissue sample. We collected demographic data (age, gender), tumor location, gross size, mitotic count, grading, cellularity type, necrosis, calculated prognostic risk, and immunohistochemical markers (CD117, CD34, DOG1) from the medical registry.

Gene expression analysis

The study focused on the gene expression analysis of DIO3 in GIST samples. We utilized FFPE gastrointestinal stromal tumor samples, including 10 control samples for comparative purposes. The RNA was isolated from these samples using Qiagen's FFPE RNA Isolation Kit (Qiagen, Crawley, UK), following the manufacturer's protocol to ensure the integrity and purity of the RNA suitable for subsequent applications. After isolation, the RNA was converted into complementary DNA (cDNA) using Qiagen's RT qPCR Kit. This reverse transcription step was performed according to the manufacturer's instructions, ensuring optimal conditions for cDNA synthesis. The target gene for this study was DIO3, with glyceraldehyde-3-phosphate dehydrogenase (GAPDH) serving as the housekeeping gene to normalize the expression levels of the target gene. Gene expression was analyzed using quantitative RT-PCR (qPCR) on the Rotor-Gene Q machine, with relative quantification. The qPCR reaction mix and cycling conditions were set according to the Qiagen RT-qPCR Kit protocol, except the recommended temperature for reading the fluorescence, which was changed from 95 °C to 60 °C, because SYBR Green gives only a signal when the ADN is double-stranded at 60°C. Each sample, including the controls, was analyzed in triplicate to ensure the reliability of the data. For data analysis, the expression levels of DIO3 were quantified relative to the housekeeping gene GAPDH using the comparative Ct (threshold cycle) method. The ΔCt value for each sample was calculated by subtracting the Ct value of GAPDH from the Ct value of DIO3. The ΔΔCt method was then used to compare the gene expression levels between the control and GIST samples. Quality control measures were strictly adhered to throughout the process. No template controls were included in each reaction to check for contamination, and technical replicates were performed to ensure the precision of the qPCR results. The amplification efficiency for each primer set was validated to ensure accurate quantification. All steps, from RNA isolation to qPCR analysis, were conducted according to the respective Qiagen kit protocols, ensuring reproducibility and accuracy of the results. This methodological approach enabled us to conduct a precise and reliable gene expression analysis of DIO3 in GIST FFPE samples. An RQ (relative quantification) value of 1 was considered normal expression, with values below 1 indicating underexpression and values above 1 indicating overexpression of the gene.

Statistical analysis

For data collection, Microsoft Excel (Microsoft Corp., Redmond, WA) and for statistical analysis, GraphPad Prism 10.2.3 (GraphPad Inc., San Diego, CA) were used. After running the Kolmogorov-Smirnov normality test, qualitative variables were expressed as frequencies and percentages, while quantitative variables were expressed as medians and interquartile ranges (IQR), respectively. For association analysis, Fisher’s exact test was performed. The confidence level was 95% and the p-value ≤0.05 was considered statistically significant.

## Results

Data from 33 patients and FFPE tissue samples were selected for DIO3 gene expression study, with a median age of 66 years (IQR 56-70), and a female/male ratio of 2/1.

The demographic, morphological, microscopical, immunohistochemical, and genetic characteristics of the GISTs are included in Table 1.

**Table 1:** Characteristics of gastrointestinal stromal tumors The values are presented as N (%) * a 50 high power field (HPF) represent an area of 5 mm2 in the optical fields used by Miettinen [20]. ** group risk in GIST was calculated considering tumor size, mitotic index and location, adapted from Miettinen et al [20]. *** necrosis was measured as the percentage of necrotic areas within the tumor. Very low-risk: size 2–5 cm, ≤ 5 mitotic index, gastric location Low-risk: size ˃5to ≤ 10 cm, ≤ 5 mitotic index, gastric location; size 2–5 cm, ≤ 5 mitotic index, intestinal location Intermediate risk: size >10 cm, ≤ 5 mitotic index, gastric location; size ˃ 5 to ≤ 10 cm, ≤ 5 mitotic index, intestinal location; size 2-5 cm, >5 mitotic index, gastric location High risk: size 2–5 cm, >5 mitotic index, intestinal location; size >10 cm, ≤ 5 mitotic index, intestinal location; size >5 to 10 cm, >5 mitotic index, gastric location; size >5 to 10 cm, >5 mitotic index, intestinal location; size >10 cm, >5 mitotic index, intestinal or gastric location

Parameters	n
Demographic data	
Total number of patients	33
Age (median, IQR)	66 (56-70) years
Female	22 (66.66%)
Male	11 (33.33%)
Tumor location	
Stomach	15 (45.46%)
Duodenum	2 (6.06%)
Small intestine	10 (30.30%)
Colon and rectum	6 (18.18%)
Gross size	
≤2 cm	4 (12.12%)
˃2 to ≤5 cm	11 (33.33%)
˃5 to≤ 10 cm	11 (33.33%)
˃10 cm	7 (21.22%)
Mitotic count (50 HPF*)	
≤ 5	21 (63.64%)
˃5	12 (36.36%)
Group risk **	
Very low/low	18 (54.55%)
Intermediate/high	15 (45.45%)
Grading	
G1	20 (60.61%)
G2	13 (39.39%)
Cell type	
Spindle	26 (78.79%
Epitheloid	2 (6.06%)
Mixed	5 (15.15%)
Necrosis***	
Absent	14 (42.42%)
˂50%	16 (48.48%)
˃50%	3 (9.1%)
Immunohistochemistry markers	
CD117	31 (93.94%)
CD34	16 (48.48%)
DOG1	21 (63.63%)
DIO3 expression	
Overexpression	23 (69.70%)
Underexpression	10 (30.30%)

In the background of our decision, the patients were classified into two risk groups (very low/low-risk and intermediate/high-risk groups) rather than individually, based on the consideration that TKI administration was carried out exactly according to this kind of classification.

The key finding was that the majority of tumors were located in the stomach (45.46%). Tumor size distribution showed that 33.33% of tumors were >2 cm to ≤5 cm and another 33.33% were >5 cm to ≤10 cm. The mitotic count revealed that 63.64% of tumors had ≤5 mitoses per 50 high-power fields (HPF). Group risk classification indicated that 54.55% of tumors were very low or low risk, while 33.33% were high risk. In terms of grading, 60.61% of tumors were G1, and 39.39% were G2. The predominant cell type was spindle (78.79%), followed by mixed (15.15%) and epitheloid (6.06%). Necrosis was absent in 42.42% of cases, less than 50% in 48.48%, and greater than 50% in 9.1% of cases.

The gender distribution depending on prognostic risk did not show significant differences (female to male ratio was 12/6 in intermediate/high-risk GISTs and 11/4 in very low/low-risk GISTs (p =0.72, Fisher’s exact test).

Immunohistochemistry markers were highly expressed, with CD117 present in 93.94% of tumors, DOG1 in 63.63%, and CD34 in 48.48%. It is noteworthy that DIO3 overexpression was observed in 69.70% of tumors, whereas underexpression was seen in 30.30% of tumors. In the two CD117-negative cases (2/33, 6.06%) DOG1 was present in 100% (2/2); in both cases, the tumors were between 2-5 cm in size, located in the gastric region, and characterized by spindle cell morphology. CD34 was present in CD117-positive tumors (16/31 cases, 51.61%), with 15/16 (93.75%) located in the gastric region and 1/16 (6.25%) located in the duodenal region. The distribution of different IHC markers was similar, in the very low/low and intermediate/high-risk GISTs, without significant differences between them. The results are presented in Table 2.

**Table 2:** Distribution of immunohistochemistry markers among gastrointestinal stromal tumors according to risk stratification The values are presented as n (%); IHC: immunohistochemistry

Risk stratification	CD117 IHC (n=31)	CD34 IHC (n=16)	DOG1 IHC (n=21)
Very low/low-risk	15 (48.38%)	8 (50%)	12 (57.14%)
Intermediate/high-risk	16 (51.62%)	8 (50%)	9 (42.86%)

Based on DIO3 gene expression, cases were divided into high DIO3 expression and low DIO3 expression groups. Our association analysis revealed no significant values in relation to patients' gender, tumor location, size, grading, mitotic count, and presence of necrosis. However, a significant association was found between DIO3 gene expression and DOG1 frequency in GISTs, a finding that could inspire future research in this area. The results are presented in Table 3.

**Table 3:** Association analysis between DIO3 expression and the different IHC markers of GISTs * statistically significant (p-value <0.05 was considered statistically significant) DIO3: deiodinase 3, IHC: immunohistochemistry, OR: odds ratio

IHC marker	DIO3 gene increased expression	DIO3 gene decreased expression	OR (95% CI)	P-value (Fisher’s exact test)
CD117 (n=31)	14 (45.16%)	17 (54.84%)	0.82 (0.04118 to 16.66)	>0.99
CD34 (n=16)	8 (50%)	8 (50%)	1.42 (0.3233 to 5.051)	0.73
DOG1 (n=21	6 (28.57%)	15 (71.83%)	0.15 (0.03616 to 0.6877)	0.02*

**Figure 1:**
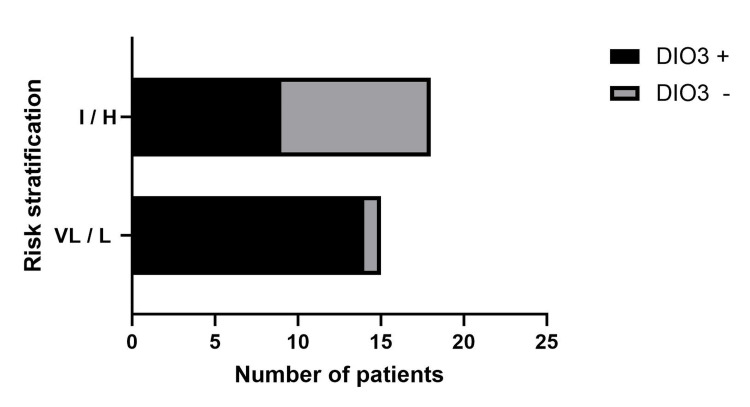
Association between DIO3 gene expression and tumor progression prognosis DIO3 +: deiodinase 3 increased expression, DIO3 -: deiodinase 3 decreased expression, I: intermediate risk, H: high risk, VL: very low risk, L: low risk

In our association analysis between DIO3 gene expression and calculated prognostic risk in GISTs, we discovered that increased gene expression in GISTs was significantly higher (OR=14 with 95% CI: 1.38 to 162.5; p˂0.009, Fisher’s exact test) in very low/low-prognostic patients. This result is depicted in Figure 1.

Given the small sample size and numerous variables, a logistic regression model was not feasible at this time. Additional research with substantial sample sizes will help clarify the role of DIO3 in the risk and prognostic stratification of the GISTs.

## Discussion

Gastrointestinal stromal tumors are the most common mesenchymal-origin tumors in the gastrointestinal tract; they generally affect men and women equally, and their frequency varies with the location within the gastrointestinal tract [21,22]. Gender distribution in GIST continues to be a topic of interest in various studies. While some sources suggest a near 1:1 male-to-female gender ratio in GIST [23], others indicate a significant male predominance [24,25]. Studies have demonstrated that gender can impact the prognosis of GIST, with the female gender being linked to a more favorable prognosis in younger patients [26]. Moreover, in some studies, male gender has been identified as an independent predictor of high pathological risk grade in GIST [27]. In contrast to this data, our study found a gender ratio of 2:1 for females and males, with no discernible differences between the genders regarding the risk.

Gastrointestinal stromal tumors are most commonly found in the stomach, accounting for approximately 60-70% of cases, followed by the small intestine with a frequency of 25-30%. GISTs in the rectum are less common, representing around 5% of cases, while those in the colon are the least frequent, constituting only about 1% of GIST occurrences [28]. In our small sample size study, we found a similar distribution of GISTs across the gastrointestinal tract.

Additionally, the frequency of GISTs with oncogenic KIT or PDGFRA mutations is high, with these mutations being present in a significant proportion of GIST cases. These mutations lead to constitutive activation of KIT kinase and are identified in a wide range of GISTs, ranging from 20% to 92% of cases [29-31]. In our study, CD117 positivity was 93.94%. Both the CD117-negative cases in our study showed DOG1 positivity, were located in the stomach, and presented spindle cell morphology. It is known that a subset of GISTs lacking CD117 mutations, known as "wild type" GISTs, accounts for approximately 10-15% of cases. DOG1 has been suggested as an alternative marker for establishing GIST diagnosis, particularly for CD117-negative GIST spindle cell tumors [32,33]. In line with this literature data, we have documented DOG1 positivity in all CD117-negative cases, while in CD117-positive cases, DOG1 was present in 19/31 (61.29%) of cases. In our study, the presence of CD34 between the used IHC markers was not specific for CD117-negative forms of GISTs or for advanced-stage tumors. It is known that CD34 has a lower sensitivity than CD117 in the IHC diagnosis of GISTs [34]. 

For risk stratification, this study used tumor location together with the size and mitotic count as suggested by Miettinen et al. [20]. However, other studies claim that only tumor size and mitotic count are sufficient for risk assessment [35]. It is essential to note that larger tumor sizes and higher mitotic activity indicate a more aggressive disease and a worse prognosis [36]. While the IHC markers did not present differences in their distribution between risk groups, high DIO3 expression was associated with the very-low/low-risk groups. Additionally, DOG1 was more frequent in the very-low/low-risk groups than in the intermediate/high-risk ones. High levels of protein DOG1 are frequently found in various types of tumors, including cancers of the esophagus, pancreas, and colon, as well as in squamous cell carcinomas from different sources. Although GISTs showed the most significant DOG1 expression, data from multiple tumor types do not support a significant prognostic role for DOG1 expression [33]. Therefore, we firmly believe that DOG1 plays a more diagnostic than prognostic role. The expression of DIO3 in GISTs in histological materials has not been directly studied previously. To the best of our knowledge, a single GIST patient was previously described with high DIO3 expression [37].

On the other hand, the results of our study underlined that the overexpression of DIO3 is not rare in GIST patients, with 69.7% of the patients showing overexpression of this gene. Elevated DIO3 activity leads to severe hyperthyrotropinemia, which does not respond to thyroxine, but only to triiodothyronine therapy. This type of hypothyroidism, namely consumptive hypothyroidism, was described after the use of TKIs in the treatment of GISTs, such as sorafenib, imatinib, or sunitinib [38,39]. However, it is important to note that increased mRNA expression does not always correlate with increased protein expression. Post-transcriptional control regulated by a lncRNA, Dio3os [40], RNA splicing, or miRNAs might also influence their clinical manifestation and therapeutic response. Nevertheless, the current study’s findings highlight the importance of assessing the DIO3 gene expression in GISTs, not only for predicting prognosis but also for establishing personalized treatment plans.

This study had several limitations, including a relatively small sample size and the lack of longitudinal follow-up data to assess long-term outcomes. Additionally, the reliance on a single-center cohort may limit the generalizability of our findings. Future research should aim to include larger, multi-center studies to validate these results and investigate the protein levels of DIO3 to fully understand its role in GISTs.

## Conclusions

This study suggests that DIO3 overexpression should be assessed before pharmacological or surgical interventions in GISTs. This is because it might have both prognostic and therapeutic roles. In patients with very low/low-risk GISTs, DIO3 overexpression might have a protective function. In intermediate/high-risk patients, it can predict side effects such as consumptive hypothyroidism, which may be associated with TKI treatment. Furthermore, large cohort studies focusing on the relationship between DIO3 expression and GIST, as well as the potential therapeutic implications, could provide valuable insights into the molecular mechanisms underlying GIST pathogenesis and treatment response. 
